# Clinical features and outcomes of atezolizumab-associated encephalitis

**DOI:** 10.3389/fimmu.2026.1943615

**Published:** 2026-10-01

**Authors:** Chunjiang Wang, Wei Sun, Liping Peng, Shaoli Zhao

**Affiliations:** 1 Department of Pharmacy, The Third Xiangya Hospital, Central South University, Changsha, Hunan, China; 2 College of Pharmacy, Changsha Medical University, Changsha, Hunan, China; 3 Department of Cardiology, The Third Xiangya Hospital, Central South University, Changsha, Hunan, China; 4 Department of Endocrinology, The Third Xiangya Hospital, Central South University, Changsha, Hunan, China

**Keywords:** atezolizumab, encephalitis, immune checkpoint inhibitors, immune-related adverse events, PD-L1 inhibitor

## Abstract

**Background:**

To explore the clinical characteristics of atezolizumab-associated encephalitis and to provide clinical evidence for diagnosis and treatment.

**Methods:**

Clinical reports on atezolizumab-associated encephalitis published before June 30, 2026 were collected for retrospective analysis by searching Chinese and English databases.

**Results:**

A total of 39 cases of encephalitis were included, with a median age of 60 years (33, 78). The median time of symptom onset was 14 days (2, 420) after the initial administration. Fever (53.8%), confusion (51.3%) and headache (28.2%) were the main complaints of the patients. Cerebrospinal fluid examination showed elevated white blood cell count and protein, with median values of 27 cells/μL (1, 553) and 133mg/dL (27.1, 815), respectively. Brain magnetic resonance imaging showed T2 fluid-attenuated inversion recovery hyperintensities in half of the patients. Electroencephalogram showed display epileptiform discharges (26.7%) and slow waves (40.0%). After discontinuation of atezolizumab and initiation of targeted treatment, 97.4% of patients achieved symptomatic improvement or recovery, while 2.6% died of encephalitis-related complications.

**Conclusion:**

Patients with atezolizumab-associated encephalitis were predominantly middle-aged and elderly, with fever, confusion and headache as the major manifestations. Clinicians should be alert to the potential risk of encephalitis during atezolizumab treatment. Most patients obtained symptom relief or clinical recovery following atezolizumab discontinuation and treatment; only a few succumbed to severe encephalitis-related complications.

## Introduction

Atezolizumab is a monoclonal antibody targeting programmed death ligand 1 (PD-L1), which selectively inhibits the interaction between PD-L1 and programmed cell death protein 1 (PD-1) on T cells, thereby relieving immune suppression and restoring the anti-tumor activity of T cells (1). Atezolizumab has been approved for the treatment of hepatocellular carcinoma (HCC), urothelial carcinoma, triple-negative breast cancer, extensive-stage small cell lung cancer, and non-small cell lung cancer (NSCLC) (2–4). Atelizumab can also attack normal organs through over-activated effector T cells, leading to immune-related adverse events (irAEs). In the III phase clinical trial of atezolizumab, the most common adverse events were cough, pyrexia, hypothyroidism, fatigue, arthralgia, pruritus, nasopharyngitis, peripheral sensory neuropathy, nausea, elevated alanine aminotransferase, and elevated aspartate aminotransferase (5, 6). Analysis of real-world pharmacovigilance data from atezolizumab indicates that encephalitis, myocarditis, hepatitis, nephritis and arthritis have the strongest risk signals (7). However, the incidence of atezolizumab-associated encephalitis is not known. The understanding of atezolizumab-associated encephalitis is mainly based on case reports, and its detailed clinical features have not yet been clarified. In the context of the increasingly widespread application of atezolizumab, it is particularly important to understand and identify the clinical characteristics of atezolizumab-associated encephalitis. This study explores the clinical features of atezolizumab-associated encephalitis, providing references for prevention, diagnosis and treatment.

## Methods

### Literature retrieval

Clinical reports of atezolizumab-associated encephalitis were collected from Chinese and English databases before June 30, 2026. The databases searched include Pubmed, embase, Web of Science, Cochrane Library, Google Scholar, China National Knowledge Infrastructure, Chinese Biomedical Literature Database, Wanfang Digital Periodicals and Chinese Science and Technology Periodicals Database. Retrieval language is Chinese and English. The search strategy was done by combining key words and subject terms (Mesh terms) with Boolean operators. Search terms included: (“atezolizumab” or “anti-PD-L1” or “PD-L1 inhibitor [s]” or “immune checkpoint inhibitor”) and (“encephalitis” or “meningoencephalitis” or”cerebellitis” or “brain inflammation”).

### Inclusion and exclusion

The included patients with atezolizumab-associated encephalitis met the diagnostic criteria for autoimmune encephalitis. Individual case reports and case series of atezolizumab-associated encephalitis were included. Repeated patients from the same institution, reviews, mechanism studies, non-atezolizumab-associated encephalitis and infectious encephalitis were excluded.

### Data extraction

Based on the self-designed form, the clinical data of the patients were extracted, including the country, gender, age, disease history, concurrent medications, tumor type, dosage, onset time, clinical symptoms, other immune-related adverse events, cerebrospinal fluid (CSF) examination, brain computerized tomography (CT), brain magnetic resonance imaging (MRI), electroencephalogram (EEG), treatment and prognosis.

### Diagnostic criteria of encephalitis

The diagnosis of immune checkpoint inhibitor (ICI)-associated encephalitis was based on the autoimmune encephalitis criteria proposed by Graus et al. Following administration of at least one dose of ICI, patients were required to meet all three of the following criteria: (1) subacute onset (rapid progression within less than 3 months) characterized by working-memory deficits (short-term memory loss), altered mental status, or psychiatric symptoms; (2) at least one of the following: new focal central nervous system manifestations, seizures in the absence of a pre-existing seizure disorder, cerebrospinal fluid white blood cell count >5 cells/μL, or brain MRI features suggestive of encephalitis; (3) reasonable exclusion of alternative etiologies that could account for the above clinical manifestations (8).

### Statistical analysis

Summary analysis of the extracted data was performed using SPSS 26. The measurement data were expressed as median values (minimum, maximum), and the count data were expressed as n (%).

## Results

### Epidemiological characteristics

After retrieving the data, a total of 35 articles containing 39 patients were included according to the inclusion and exclusion criteria. The basic information of the 39 patients is summarized in **Table 1**. The median age of the 39 patients was 60 years (33, 78), including 20 (52.6%) males. The countries with the most reports were Japan (13 cases, 33.3%) and South Korea (8 cases, 20.5%). The median time to the onset of encephalitis was 14 days (2, 420) after the initial administration of atezolizumab, and the median cycle was 1 cycle (1, 47). The types of these patients were lung cancer (17 cases, 43.6%), hepatocellular carcinoma (8 cases, 20.5%), bladder cancer (7 cases, 18.0%), and breast cancer (6 cases, 15.4%) and cervical cancer (1 case, 2.6%). Ten (25.6%) patients had a history of diseases, such as diabetes, hypertension, and ischemic cardiopathy. Nineteen (48.7%) patients concurrently used other drugs, such as bevacizumab, carboplatin, etoposide, cisplatin, and nab-paclitaxel.

**Table 1:** Epidemiological characteristics of 39 patients with encephalitis.

Parameter	Classification	Value
Sex (38)^a^	male	20 (52.6%)
	female	18 (47.4%)
Age (37)^a^	years	60 (33, 78) ^b^
Country	Japan	13 (33.3%)
	Korea	8 (20.5%)
	USA	5 (12.8%)
	China	4 (10.3%)
	France	3 (7.7%)
	Germany	1 (2.6%)
	Lebanon	1 (2.6%)
	Pakistan	1 (2. 6%)
	Russia	1 (2. 6%)
	Thailand	1 (2. 6%)
	United Kingdom	1 (2. 6%)
Onset time	First infusion to onset of encephalitis	
	days (33)^a^	14 (2, 420) ^b^
	cycle (33)^a^	1 (1, 47) ^b^
Dosage (12)^a^	1200 mg	11 (91.7%)
	840 mg	1 (8.83%)
Cancers	lung cancer	17 (43.6%)
	hepatocellular carcinoma	8 (20.5%)
	bladder cancer	7 (18.0%)
	breast cancer	6 (15.4%)
	cervical cancer	1 (2.6%)
Medical history	chronic hepatitis B, hepatitis C, dyslipidemia, diabetes, hypertension, ischemic cardiopathy, renal insufficiency, rheumatoid arthritis, liver cirrhosis, depression, pigmentary degeneration of the retina	10 (25.6%)
Concomitant Medications	bevacizumab, carboplatin, etoposide, cisplatin, nab-paclitaxel, tamoxifen, rosuvastatin, amlodipine, benidipine, carvedilol, candesartan, cobimetinib, fulvestrant, ipatasertib	19 (48.7%)

a Represents the number of patients with this parameter out of 39 patients.

b Median (minimum-maximum).

### Clinical manifestation

The clinical manifestations of the 39 patients are summarized in **Table 2**. Fever (21 cases, 53.8%), confusion (20 cases, 51.3%), cognitive decline (12 cases, 30.8%) and headache (11 cases, 28.2%) were the most common symptoms. Other symptoms included seizure (9 cases, 23.1%), gait disturbance (6 cases, 15.4%), altered mental status (6 cases, 15.4%), aphasia (5 cases, 12.8%), drowsiness (5 cases, 12.8%), bradykinesia (5 cases, 12.8%), fatigue (4 cases, 10.3%), coma (4 cases, 10.3%), vomiting (4 cases, 10.3%), disorientation (4 cases, 10.3%) and poor appetite (3 cases, 10.3%). Nine (23.1%) patients developed other irAEs, such as Guillain–Barre syndrome, acute motor and sensory axonal neuropathy, colitis, thrombocytopenia, rash, and polyneuropathy.

**Table 2:** Clinical manifestations and laboratory examination of 39 patients with encephalitis.

Parameter	Classification	Value
Symptom	fever	21 (53.8%)
	confusion	20 (51.3%)
	cognitive decline	12 (30.8%)
	headache	11 (28.2%)
	seizure	9 (23.1%)
	altered mental status	6 (15.4%)
	bradykinesia	6 (15.4%)
	aphasia	5 (12.8%)
	drowsiness	5 (12.8%)
	bradykinesia	5 (12.8%)
	fatigue	4 (10.3%)
	coma	4 (10.3%)
	vomiting	4 (10.3%)
	disorientation	4 (10.3%)
	poor appetite	3 (7.7%)
	nausea	2 (5.1%)
	respiratory failure	2 (5.1%)
	dysarthria	2 (5.1%)
	emotional lability	2 (5.1%)
	dysphagia	1 (2.6%)
	diplopia	1 (2.6%)
	restlessness and delirium	1 (2.6%)
	irritability	1 (2.6%)
	weakness	1 (2.6%)
	dizziness	1 (2.6%)
	hypothermia	1 (2.6%)
Other irAEs	GBS, AMSAN, colitis, thrombocytopenia, rash, polyneuropathy, hepatitis, pneumonitis, myelitis, mucositis, eosinophilia, cranial nerve palsies	9 (23.1%)
CSF (35)^a^		
White blood cells (33) ^a^	normal	3 (9.1%)
	leukocytosis	30 (90.9%)
	neutrophils predominate	8 (26.7%)
	lymphocytosis predominate	7 (23.3%)
	monocytosis predominate	5 (16.7%)
	nr	10 (32.3%)
	cells/μL	27 (1, 553) ^b^
Protein (35) ^a^	normal	3 (8.6%)
	elevated	32 (91.4%)
	mg/dL	133 (27.1, 815) ^b^
Glucose (30) ^a^	normal	19 (63.3%)
	elevated	11 (36.7%)
	mg/dL	77 (44, 124) ^b^
Cytology (15) ^a^	negative	15 (100%)
Oligoclonal bands (10)^a^	negative positive	5 (50.0%) 5 (50.0%)
White blood cell (10)^a^	× 10^9^/L	4.4 (2.1, 10) ^b^
C-reactive protein (9)^a^	mg/dL	6 (0.4, 54) ^b^
Antibodies (28)^a^	negative	18 (64.3%)
	positive	10 (35.7%)
	anti-Hu	6 (60.0%)
	anti-SOX1	2 (20.0%)
	anti- CV2	1 (10.0%)
	anti- Zic4	1 (10.0%)
	anti- GABABR	1 (10.0%)
	anti- CAMKV	1 (10.0%)

irAEs, immune-related adverse events; CAMKV, calmodulin kinase-like vesicle-associated; SOX1, Sry-like high mobility group box 1; GABABR, gamma-amino butyric acid B receptor; anti-CV2, collapsin response mediator protein 5; anti-Zic4, zinc finger protein of the cerebellum 4; AMSAN, acute motor and sensory axonal neuropathy; GBS, Guillain–Barre syndrome; nr, not reported.

a Represents the number of patients with this parameter out of 39 patients.

b Median (minimum-maximum).

### Cerebrospinal fluid examination

CSF examinations were performed on 35 patients. CSF white blood cell counts were available for 33 patients, with a median of 27 cells/μL (1, 553). Thirty of 33 patients exhibited CSF pleocytosis, including neutrophilic (8, 26.7%), lymphocytic (7, 23.3%), and monocytic predominance (5, 16.7%). Protein was described in 35 patients, with a median value of 133 mg/dL (27.1, 815). Of the 30 patients who underwent CSF glucose testing, 19 presented with normal glucose levels, and the median glucose value was 77 mg/dL (44, 124). Oligoclonal bands were identified in 10 patients, and 5 (50.0%) of these patients showed positive results. Fifteen patients described CSF cytology, which showed no malignant cells.

### Encephalitis related antibodies

Antibodies were described in 28 patients, of whom 18 (64.3%) were negative. Another 10 (35.7%) patients showed positive results, presenting as anti-Hu antibodies (6 cases, 60.0%), anti-SOX1 antibodies (2 cases, 20.0%), anti-CV2 antibodies (1 case, 10.0%), and anti-Zic4 antibodies (1 case, 10.0%), anti-GABABR antibodies (1 case, 10.0%) and anti-CAMKV antibodies (1 case, 10.0%).

### Laboratory tests

Blood white blood cells were described in 10 patients, with a median value of 4.4 × 10^9^/L (2.1, 10). C-reactive protein was described in 9 patients, with a median value of 6 mg/dL (0.4, 54).

### Head imaging examination

The summary of the head imaging examinations for 39 patients is presented in **Table 3**. Brain MRI examinations were conducted on 36 patients, and 10 (27.8%) of them showed no abnormalities. The remaining 26 patients mainly showed T2/fluid-attenuated inversion recovery (FLAIR) hyper-signal (18 cases, 50.0%) and leptomeningeal enhancement (5 cases, 13.8%). Brain CT was performed in 7 patients, of which 6 (85.7%) patients showed no abnormalities, and only 1 (14.3%) patient had subdural hematoma and subarachnoid hemorrhage.

**Table 3:** Brain imaging examination of 39 patients with encephalitis.

Parameter	Classification	Value
MRI (36)^a^	normal	10 (27.8%)
	T2/FLAIR hypersignal	18 (50.0%)
	leptomeningeal enhancement	5 (13.8%)
	cerebral infarction	1 (2.8%)
	enhancing lesion	1 (2.8%)
	hematoma and hemorrhage	1 (2.8%)
EEG (15)^a^	normal	5 (33.3%)
	epileptiform discharges	4 (26.7%)
	slow waves	6 (40.0%)
CT (7)^a^	normal	6 (85.7%)
	hematoma and hemorrhage	1 (14.3%)
Classification of encephalitis (11) ^a^	limbic encephalitis	7 (63.6%)
	striatal encephalitis	2 (18.2%)
	meningoencephalitis	1 (9.1%)
	BBE	1 (9.1%)

BBE, bickerstaff brainstem encephalitis; CT, computerized tomography; EEG, electroencephalography; FLAIR, fluid attenuation inversion recovery; MRI, magnetic resonance imaging.

### Electroencephalography

EEG examinations were conducted on 15 patients, and 5 of them showed no abnormalities. The remaining 10 patients could display epileptiform discharges (4 cases, 26.7%) and slow waves (6 cases, 40.0%).

### Classification of encephalitis

Eleven patients reported the types of encephalitis, including limbic encephalitis (7 cases, 63.6%), striatal encephalitis (2 cases, 18.2%), meningoencephalitis (1 case, 9.1%), and Bickerstaff brainstem encephalitis (1 case, 9.1%).

### Treatment and prognosis

The treatment and prognosis of 39 patients are presented in **Table 4**. All patients withdrew atezolizumab and received steroid therapy. On the basis of steroid treatment, 14 (35.9%) patients received intravenous immunoglobulin (IVIG), 6 (15.4%) patients received plasma exchange (PLEX), and 3 (7.7%) patients received rituximab or tocilizumab. Twelve (30.8%) patients received empirical antibiotic therapy. After treatment, the symptoms of 38 (97.4%) patients were improved or recovered, and 1 (2.6%) had no improvement. Of the four fatal cases, 1 (25.0%) succumbed to encephalitis complications and 3 (75.0%) to cancer progression.

**Table 4:** Treatment and outcome of 39 patients with encephalitis.

Parameter		Parameter
Treatment	withdraw	39 (100%)
	steroids	21 (53.8%)
	steroids+ IVIG	10 (25.6%)
	steroids + PLEX	4 (10.3%)
	steroids + IVIG + PLEX	1 (2.6%)
	steroids + IVIG + PLEX + rituximab	1 (2.6%)
	steroids + IVIG + rituximab/tocilizumab	2 (5.1%)
	empiric antibiotics	12 (30.8%)
Outcome	improvement or recovery	38 (97.4%)
	no improvement	1 (2.6%)
	death	4 (10.3%)
	encephalitis	1 (25.0%)
	cancer progression	3 (75.0%)

IVIG, intravenous immunoglobulin; PLEX, plasmapheresis.

## Discussion

Neurologic adverse events occurred in less than 1% of patients treated with ICI, and autoimmune encephalitis accounted for 0.2%-0.5% of all neurological adverse events (9, 10). Analysis from the Japanese Adverse Drug Event Report (JADER) database found that encephalitis occurred more frequently with atezolizumab, pembrolizumab, nivolumab, and ipilimumab compared with other ICIs (11). According to the OAK III phase randomized controlled trial, the incidence of encephalitis in patients with non-small cell lung cancer after treatment with atezolizumab was only 0.8% (3). In an open-label Phase III trial, only one of the 264 patients with small cell lung cancer who received chemoradiation + atezolizumab treatment developed encephalitis (12). Data from the real world indicate that men are more likely to experience adverse neurological events when using ICI. The male and female patients are mainly elderly (13, 14). The median age of atezolizumab-associated encephalitis was 60 years old, with slightly more males (53%) than females (47%). Whether age affects the efficacy and safety of ICI has drawn people’s attention (15). Although most studies have shown that the efficacy and safety of ICI are not related to age, age should still be taken into account when using ICI therapy. These patients mainly come from Asia, followed by Europe and America, indicating a wide geographical distribution.

Atezolizumab-associated encephalitis can only be diagnosed after all causes of encephalitis have been carefully ruled out. atezolizumab-associated encephalitis can present with a variety of clinical symptoms that are nonspecific, and common symptoms include fever, confusion, headache, and seizure. Encephalitis usually occurs 2 weeks after the first administration cycle, or it can occur 420 days after the initial administration. This indicates that the timing of the onset of encephalitis varies greatly and is difficult to predict. The sensitivity of MRI is relatively low, with approximately 28% of patients showing no abnormalities on MRI at the time of onset. About half of the patients showed increased signal intensity on T2-weighted imaging and FLAIR sequences. Almost all of the CSF has inflammatory changes, manifested as elevated cell counts and proteins. The white blood cells in CSF can be dominated by neutrophils, lymphocytes, or monocytes. Paraneoplastic antibodies are associated with various neurological disorders, and their presence may increase the risk of encephalitis (16). Immunoglobulins are present at low physiological concentrations in the CSF of healthy individuals owing to physiologic diffusion across the blood-CSF barrier, whereas encephalitis may result in the appearance of CSF-restricted oligoclonal bands (17). Similarly, in the CSF of patients with atezolizumab-associated encephalitis, an oligoclonal band can be observed.

Paraneoplastic neurological syndromes are most frequently observed in patients with small cell lung cancer, breast cancer, gynecological tumors, and hematological malignancies (18). A previous systematic review of ICI-related encephalitis reported negative paraneoplastic antibodies in 48% of affected patients (19). In line with these findings, our cohort revealed detectable paraneoplastic antibodies in 32% of patients with atezolizumab-associated encephalitis. Notably, intracellular paraneoplastic antibodies were far more prevalent than surface antibodies in our cases, strongly suggesting an underlying paraneoplastic autoimmune predisposition in this patient population. Positive paraneoplastic serology generally indicates pre-existing tumor-driven autoimmunity, which can be further amplified and clinically unmasked by ICI-mediated immune activation. However, retrospective case-based data cannot reliably clarify whether these autoantibodies predated atezolizumab exposure or arose secondarily following ICI-related immune perturbation. Individual reassessment of cases harboring high-risk paraneoplastic antibodies, including anti-Hu and CRMP5/CV2, further confirmed that these neuroimmunological manifestations cannot be uniformly defined as definite atezolizumab-induced encephalitis. Some cases presented typical clinical features of classic malignancy-driven paraneoplastic neurological syndromes, which were temporally coincident rather than causally linked to atezolizumab treatment. In other cases, the rapid onset of neurological symptoms shortly after atezolizumab initiation primarily reflected ICI-triggered unmasking of subclinical pre-existing paraneoplastic autoimmunity, where immune checkpoint inhibition disrupted immune tolerance and precipitated overt neurological injury. Moreover, several cases exhibited an overlap phenotype that integrated characteristic features of both ICI-related neurotoxicity and paraneoplastic autoimmunity. Due to the absence of standardized pretreatment antibody screening and prospective longitudinal immune monitoring in published cases, these distinct etiological mechanisms remain indistinguishable in our retrospective analysis.

The exact mechanism of atezolizumab-associated encephalitis remains uncertain. Firstly, the anti-tumor immune response triggered by ICI may be related to cross-reactivity with the self-antigens of the central nervous system. Secondly, ICI may enhance the pre-existing immune responses against tumors and the nervous system. Thirdly, ICI can recognize PD-1, PD-L1 and CTLA4 on intrinsic cells within the nervous system such as endothelial cells and astrocytes, thereby directly causing local damage through complement-dependent or cell-dependent cytotoxic mechanism (20, 21). PD-L1 is highly expressed in astrocytes and microglia. PD-1 inhibitors or PD-L1 inhibitors that block the interaction between PD -L1 and PD-1 on lymphocytes infiltrating the central nervous system may aggravate local inflammation or bring potential central nervous system inflammation to the surface (22). Furthermore, genetic susceptibility may have been involved in ICI-related encephalitis. According to a report from South Korea, HLA-B*27:05 might be a genetic factor that increases the risk of atezolizumab-associated encephalitis (23). In conclusion, the pathophysiological mechanism of atezolizumab-associated encephalitis requires further exploration.

There is no optimal treatment for ICI-associated encephalitis. According to the severity, ICI-associated encephalitis is classified into grades G1 to G5. For different grades, patients must immediately stop ICI treatment and receive drug therapy at an early stage (24–26). Steroids remain the first-line treatment drugs. For G1 grade patients, it is recommended to take prednisone orally at a dose of 0.5 to 1 mg/kg/day for a short period of time. For G2 grade patients, it is recommended to administer intravenous methylprednisolone at a dose of 1–2 mg/kg/day. For patients with grade G2 or above, intravenous methylprednisolone (1 g/day for 3–5 days) combined with IVIG or PLEX is recommended. For refractory patients, immunosuppressants such as rituximab, infliximab, and mycophenolate mofetil can be considered. Of the 39 patients in this study, 97.4% improved after aggressive treatment, and only 2.6% died. However, the sample size was too small to truly reflect the treatment of atezolizumab-associated immune-related encephalitis. Whether ICI can be used for treatment again after the occurrence of ICI-related encephalitis is a key issue of clinical concern. The current guidelines recommend that patients with grade G2-G4 encephalitis permanently discontinue ICI. A study involving 40 patients with multiple tumor types who received ICI re-treatment found that up to 42.5% of the patients experienced recurrence of the same type of irAEs, and 12.5% of the patients experienced different types of irAEs (27). In another retrospective analysis, it was found that among 38 patients with NSCLC who received ICI retreatment, 26% of them developed recurrent irAEs and new-onset irAEs, respectively (28). None of the patients with atezolizumab-associated encephalitis restarted atezolizumab treatment. Further clinical research is needed to clarify the standards for re-challenging ICI.

### Limitations

Several limitations of the article need to be taken into consideration. First, the enrolled patients were predominantly obtained from case reports, which gives rise to publication bias; specifically, severe or rare cases are more likely to be reported. Given the lack of a control group, we could not perform variable-level analyses for risk factors. Second, not all patients provided complete clinical data, which may lead to bias and affect the accuracy of the research results. Third, this study did not compare atezolizumab-associated encephalitis with encephalitis induced by other PD-L1 inhibitors. Therefore, itis not yet possible to determine whether atezolizumab-associated encephalitis has unique clinical characteristics. Fourth, not all the included patients had the uniformly collected pretreatment baseline neuronal/paraneoplastic antibody results. This inherent diagnostic ambiguity precludes fully unambiguous etiologic attribution for a small proportion of included cases, and this uncertainty should be considered when interpreting the reported causal associations between atezolizumab and encephalitis. Future prospective cohort studies with systematic pretreatment antibody screening and longitudinal immunophenotyping will be required to reliably disentangle these clinically overlapping entities. Fifth, the author has not discussed whether atezolizumab should be restarted after the encephalitis has been alleviated. Despite these multiple limitations, the present study still carries clinical value. It provides references for clinicians regarding the diagnosis and intervention planning of atezolizumab-associated encephalitis.

## Conclusion

Atezolizumab-associated encephalitis is rare and fatal. As the indications for atezolizumab expand, it is crucial for clinicians to recognize atezolizumab-associated encephalitis. atezolizumab-associated encephalitis usually occurs 14 days after the first administration cycle and has no specific neurological symptoms. Many questions remain to be addressed in atezolizumab-associated encephalitis, such as risk factors, pathogenesis, optimal treatment, and restarting after drug discontinuation.

## Data Availability

The original contributions presented in the study are included in the article/**Supplementary Material**. Further inquiries can be directed to the corresponding author.

## Supplementary material

The Supplementary Material for this article can be found online at: https://www.frontiersin.org/articles/10.3389/fimmu.2026.1943615/ full#supplementary-material
