# Supplementary material for: Clinical features and outcomes of atezolizumab-associated encephalitis
Source: Front Immunol. 2026 Oct 1;17:1943615. doi: 10.3389/fimmu.2026.1943615 (PMC13668502; doi:10.3389/fimmu.2026.1943615)
Supplement: Supplementary file 1 [file Table1.docx]

Supplementary Table S1 Clinical data of 39 patients with atezolizumab-associated encephalitis

| Ref | sex/age | Cancers | onset time（days） | cycles | antibodies | CSF:glucose( mg/dL) | CSF:protein( mg/dL) | CSF:white Cell count(/μL) | MRI | EEG | treatment | outcome |
| --- | --- | --- | --- | --- | --- | --- | --- | --- | --- | --- | --- | --- |
| 1 | F/59 | bladder cancer | 12 | 1 | negative | 80 | 100 | normal | enhancing lesion | nr | D,steroids,EA | Recovery |
| 2 | F/53 | Cervical cancer | 13 | 1 | negative | 92 | >600 | 553 | diffuse leptomeningeal enhancement | epileptiform discharges | D,steroids,EA | improvement |
| 3 | M/78 | lung cancer | 13 | 1 | negative | elevated | 132 | 139 | normal | nr | D,steroids,EA | improvement |
| 4 | M/49 | bladder cancer | 14 | 1 | negative | 124 | 218.1 | 59 | leptomeningeal enhancement | normal | D,IVIG ,EA | recovery |
| 5 | M/71 | lung cancer | 56 | 3 | anti-Hu | nr | 78 | 32 | normal | nr | D,IVIG | improvement |
| 6 | F/48 | lung cancer | 13 | 1 | nr | elevated | elevated | 62 | T2/ FLAIR hypersignal | normal | D,steroids,EA | improvement |
| 7 | M/56 | lung cancer | 17 | 1 | negative | 73 | 166 | 20 | normal | nr | D,steroids | improvement |
| 8 | M/76 | lung cancer | nr | 4 | anti-CRMP5 | normal | 76.4 | 12 | T2/ FLAIR hypersignal | nr | D,steroids | improvement |
| 9 | F/72 | lung cancer | 270 | nr | negative | nr | nr | nr | T2/ FLAIR hypersignal | nr | D,IVIG | improvement |
| 10 | F/38 | breast cancer | 10 | 1 | nr | 79 | 60 | 15 | T2/ FLAIR hypersignal | nr | D,steroids,EA | improvement |
| 11 | F/70 | HCC | 10 | 1 | negative | 71 | 549.4 | 179 | normal | nr | D, PLEX,EA | improvement |
| 12 | M/48 | lung cancer | 16 | 1 | negative | 50 | 162 | 12 | T2/ FLAIR hypersignal | nr | D,steroids,EA | improvement |
| 13 | M/69 | lung cancer | 60 | nr | anti-Hu and anti-CV2 | 58 | 44 | 20 | normal | slow waves | D,steroids, IVIG | improvement |
| 14 | M/66 | lung cancer | 60 | 3 | anti-Hu and anti-Zic4 | 72 | 94 | 5 | T2/ FLAIR hypersignal | nr | D,steroids, IVIG | improvement |
| 15 | F/42 | HCC | 12 | 1 | nr | 82 | 236 | 514 | T2/ FLAIR hypersignal | nr | D,steroids | improvement |
| 16 | F/65 | breast cancer | nr | 4 | nr | nr | nr | nr | T2/ FLAIR hypersignal | nr | D,steroids | death |
| 17 | F/71 | lung cancer | 120 | nr | nr | 62 | 447 | 510 | normal | normal | D,steroids | Recovery |
| 18 | M/76 | HCC | 14 | 1 | nr | 121 | 815 | 27 | hematoma and hemorrhage | nr | D,steroids | Recovery |
| 19 | M/58 | bladder cancer | nr | 47 | negative | 64.1 | 40.6 | 2 | T2/ FLAIR hypersignal | nr | D,steroids | improvement |
| 20 | M/nr | bladder cancer | 14 | 1 | anti-CAMKV | nr | 60 | 8 | T2/ FLAIR hypersignal | epileptiform discharges | D,steroids, PLEX | Recovery |
| 21 | nr/nr | lung cancer | 2 | 1 | negative | nr | 53.2 | 1 | normal | normal | D,steroids, IVIG | improvement |
| 22 | M/45 | HCC | 10 | 1 | nr | 115 | > 600 | 52 | leptomeningeal enhancement | normal | D,steroids,EA | improvement |
| 23 | M/65 | HCC | 16 | 1 | negative | 72 | 146 | 7 | normal | nr | D,steroids,EA | improvement |
| 24 | F/60 | breast cancer | 10 | 1 | negative | 115 | 466 | nr | leptomeningeal enhancement | epileptiform discharges | D,steroids, IVIG | improvement |
| 25 | M/67 | lung cancer | nr | 1 | anti-GABABR | nr | 660 | nr | T2/ FLAIR hypersignal | epileptiform discharges | D,steroids, IVIG,PLEX,rituximab, tocilizumab | improvement |
| 26 | F/33 | breast cancer | nr | nr | negative | 72.07 | 610 | 6 | T2/ FLAIR hypersignal | nr | D,steroids, IVIG,PLEX | improvement |
| 27 | F/63 | lung cancer | 41 | 2 | anti-Hu | normal | 95 | 16 | T2/ FLAIR hypersignal | nr | D,steroids, IVIG | improvement |
| 28 | M/66 | HCC | 8 | 1 | nr | 68 | >500 | 190 | nr | nr | D,steroids, EA | improvement |
| 29 | M/60 | lung cancer | 420 | nr | Hu-Abs | 64 | 27.1 | 27 | T2/ FLAIR hypersignal | slow waves | D,steroids | improvement |
| 30 | M/47 | lung cancer | nr | nr | Hu-Abs | nr | nr | nr | nr | nr | D,steroids | improvement |
| 31 | F/56 | lung cancer | 107 | 6 | nr | nr | 100 | 10 | T2/ FLAIR hypersignal | nr | D,steroids, PLEX | improvement |
| 32 | M/68 | HCC | 13 | 1 | nr | nr | nr | nr | normal | nr | D,steroids | improvement |
| 33 | M/68 | HCC | 14 | 1 | negative | 44 | 159 | 8 | cerebral infarction | nr | D,steroids | improvement |
| 34 | F/66 | lung cancer | 14 | 1 | anti-SOX1 | 84.69 | 122 | nr | nr | nr | D,steroids, PLEX,EA | improvement |
| 35 | F/37 | breast cancer | 15 | 1 | negative | 76 | nr | 54 | leptomeningeal enhancement | nr | D,steroids, IVIG | improvement |
| 35 | F/53 | bladder cancer | 18 | 1 | negative | 81 | nr | 222 | T2/ FLAIR hypersignal | slow waves | D,steroids, IVIG,rituximab, tocilizumab | improvement |
| 35 | F/70 | bladder cancer | 15 | 1 | negative | 79 | nr | 30 | T2/ FLAIR hypersignal | slow waves | D,steroids, IVIG,rituximab | improvement |
| 35 | M/42 | bladder cancer | 15 | 1 | negative | 78 | nr | 65 | normal | slow waves | D,steroids, IVIG | improvement |
| 35 | F/60 | breast cancer | 16 | 1 | negative | 86 | nr | 135 | T2/ FLAIR hypersignal | slow waves | D,steroids | improvement |

D:Discontinuation; EA,empiric antibiotics;nr,not reported; HCC, hepatocellular carcinoma;IVIG, intravenous immunoglobulin; PLEX, plasmapheresis.

References

1. Levine JJ, Somer RA, Hosoya H, Squillante C. Atezolizumab-induced Encephalitis in Metastatic Bladder Cancer: A Case Report and Review of the Literature. Clin Genitourin Cancer. 2017 Oct;15(5):e847-e849. doi: 10.1016/j.clgc.2017.03.001.
2. Laserna A, Tummala S, Patel N, El Hamouda DEM, Gutiérrez C. Atezolizumab-related encephalitis in the intensive care unit: Case report and review of the literature. SAGE Open Med Case Rep. 2018 Aug 2;6:2050313X18792422. doi: 10.1177/2050313X18792422.
3. Arakawa M, Yamazaki M, Toda Y, Saito R, Ozawa A, Kosaihira S, Kimura K. Atezolizumab-induced encephalitis in metastatic lung cancer: a case report and literature review. eNeurologicalSci. 2018 Dec 17;14:49-50. doi: 10.1016/j.ensci.2018.12.001.
4. Kim A, Keam B, Cheun H, Lee ST, Gook HS, Han MK. Immune-Checkpoint-Inhibitor-Induced Severe Autoimmune Encephalitis Treated by Steroid and Intravenous Immunoglobulin. J Clin Neurol. 2019 Apr;15(2):259-261. doi: 10.3988/jcn.2019.15.2.259.
5. Mongay-Ochoa N, Vogrig A, Muñiz-Castrillo S, Honnorat J. Anti-Hu-associated paraneoplastic syndromes triggered by immune-checkpoint inhibitor treatment. J Neurol. 2020 Jul;267(7):2154-2156. doi: 10.1007/s00415-020-09940-y.
6. Robert L, Langner-Lemercier S, Angibaud A, Sale A, Thepault F, Corre R, Lena H, Ricordel C. Immune-related Encephalitis in Two Patients Treated With Immune Checkpoint Inhibitor. Clin Lung Cancer. 2020 Sep;21(5):e474-e477. doi: 10.1016/j.cllc.2020.03.006.
7. Yamaguchi Y, Nagasawa H, Katagiri Y, Wada M. Atezolizumab-associated encephalitis in metastatic lung adenocarcinoma: a case report. J Med Case Rep. 2020 Jul 4;14(1):88. doi: 10.1186/s13256-020-02411-y.
8. Tatsumi S, Uryu K, Iwasaki S, Harada H. A Case of Anti-CRMP5 Paraneoplastic Neurological Syndrome Induced by Atezolizumab for Small Cell Lung Cancer. Intern Med. 2020 Aug 12. doi: 10.2169/internalmedicine.4889-20. Epub ahead of print. PMID: 32788536.
9. Nishijima H, Suzuki C, Kon T, Nakamura T, Tanaka H, Sakamoto Y, Tomiyama M. Bilateral Thalamic Lesions Associated With Atezolizumab-Induced Autoimmune Encephalitis. Neurology. 2021 Jan 19;96(3):126-127. doi: 10.1212/WNL.0000000000011297.
10. Nader R, Tannoury E, Rizk T, Ghanem H. Atezolizumab-induced encephalitis in a patient with metastatic breast cancer: a case report and review of neurological adverse events associated with checkpoint inhibitors. Autops Case Rep. 2021 Apr 15;11:e2021261. doi: 10.4322/acr.2021.261.
11. Özdirik B, Jost-Brinkmann F, Savic LJ, Mohr R, Tacke F, Ploner CJ, Roderburg C, Müller T. Atezolizumab and bevacizumab-induced encephalitis in advanced hepatocellular carcinoma: Case report and literature review. Medicine (Baltimore). 2021 Jun 18;100(24):e26377. doi: 10.1097/MD.0000000000026377.
12. Watanabe S, Yoshizumi K, Moriguchi S, Higashiyama T, Negi Y, Mikami K, Kimura T. Nivolumab treatment followed by atezolizumab induced encephalitis and neuropathy with antiganglioside antibodies. eNeurologicalSci. 2021 Dec 3;25:100386. doi: 10.1016/j.ensci.2021.100386.
13. Wada S, Iwamoto K, Ozaki N. Atezolizumab, an immune checkpoint inhibitor, caused precedent depressive symptoms related to limbic encephalitis. Psychiatry Clin Neurosci. 2022 Apr;76(4):125-126. doi: 10.1111/pcn.13325.
14. Nakashima K, Demura Y, Kurokawa K, Takeda T, Jikuya N, Oi M, Tada T, Akai M, Ishizuka T. Immune Checkpoint Inhibitor-Induced Limbic Encephalitis during Treatment with Atezolizumab in a Patient with Small-Cell Lung Cancer: A Case Report and Review of the Literature. Case Reports Immunol. 2022 Jan 6;2022:9290922. doi: 10.1155/2022/9290922.
15. Satake T, Maruki Y, Kubo Y, Takahashi M, Ohba A, Nagashio Y, Kondo S, Hijioka S, Morizane C, Ueno H, Okusaka T. Atezolizumab-induced Encephalitis in a Patient with Hepatocellular Carcinoma: A Case Report and Literature Review. Intern Med. 2022;61(17):2619-2623. doi: 10.2169/internalmedicine.8919-21.
16. Chen G, Zhang C, Lan J, Lou Z, Zhang H, Zhao Y. Atezolizumab-associated encephalitis in metastatic breast cancer: A case report. Oncol Lett. 2022 Jul 27;24(3):324. doi: 10.3892/ol.2022.13444.
17. Ibrahim EY, Zhao WC, Mopuru H, Janowiecki C, Regelmann DJ. Hope, Cure, and Adverse Effects in Immunotherapy: Atezolizumab-Associated Encephalitis in Metastatic Small Cell Lung Cancer - A Case Report and Literature Review. Case Rep Neurol. 2022;14(3):366-371. doi: 10.1159/000526248.
18. Chao KH, Tseng TC. Atezolizumab-induced encephalitis with subdural hemorrhage and subarachnoid hemorrhage in a patient with hepatocellular carcinoma. J Formos Med Assoc. 2023;122(11):1208-1212. doi: 10.1016/j.jfma.2023.07.019.
19. Prasertpan T, Teeyapun N, Bhidayasiri R, Sringean J. Immune Checkpoint Inhibitor-Associated Striatal Encephalitis Presenting with Subacute Progressive Parkinsonism. Mov Disord Clin Pract. 2023 Aug 24;10(Suppl 3):S7-S11. doi: 10.1002/mdc3.13800.
20. Gilligan M, Lesnick CE, Guo Y, Bradshaw MJ, Ladha SS, Nowak M, Shah MP, Wittenborn JR, Basal E, Hinson S, Yang B, Dubey D, Mills JR, Pittock SJ, Zekeridou A, McKeon A. Paraneoplastic Calmodulin Kinase-Like Vesicle-Associated Protein (CAMKV) Autoimmune Encephalitis. Ann Neurol. 2024 Jul;96(1):21-33. doi: 10.1002/ana.26943.
21. Buckley MW, Balaji Warner A, Brahmer J, Cappelli LC, Sharfman WH, Fuchs E, Kang H, Forde PM, Gladstone DE, Ambinder R, Kelly RJ, Lipson EJ, Gojo I, Lee EJ, Johnson TP, Saidha S, Llinas R, Ostrow LW, Naidoo J, Probasco JC. Immune-related encephalitis after immune checkpoint inhibitor therapy. Oncologist. 2025 Jan 17;30(1):oyae186. doi: 10.1093/oncolo/oyae186.
22. Sagong M, Kim KT, Jang BK. Atezolizumab- and bevacizumab -induced encephalitis in a patient with advanced hepatocellular carcinoma: a case report and literature review. J Cancer Res Clin Oncol. 2024 Aug 24;150(8):397. doi: 10.1007/s00432-024-05918-9.
23. Otomo K, Fujita M, Sekine R, Sato H, Abe N, Sugaya T, Watanabe C, Takahata Y, Hayashi M, Abe K, Takahashi A, Ohira H. Patient with Atezolizumab-induced Encephalitis in Hepatocellular Carcinoma. Intern Med. 2025 Apr 15;64(8):1181-1187. doi: 10.2169/internalmedicine.4321-24.
24. Shimoyama K, Nakajima A, Minari Y. The First Report of Bickerstaff Brainstem Encephalitis Induced by Atezolizumab for Metastatic Breast Cancer. Acta Med Okayama. 2024 Oct;78(5):407-412. doi: 10.18926/AMO/67665.
25. Cezar M, Belletto R, Polidori S, Soive L, Campitiello M, Legros PO, Egea J, Plastino F, Benaiteau M, Tyvaert L, Tortora G, Longo R. Managing paraneoplastic GABABR-related limbic encephalitis in a patient with a small cell lung cancer receiving an Immune-Checkpoint Inhibitor (ICI) therapy: description of a case report. BMC Neurol. 2025 Nov 14;25(1):467. doi: 10.1186/s12883-025-04483-z.
26. Wahab A, Faraz A. Atezolizumab Immunotherapy-Induced Encephalitis in a Patient With Triple-Negative Breast Cancer: A Case Report. Cureus. 2025 Dec 16;17(12):e99387. doi: 10.7759/cureus.99387.
27. Li Z, Wang H. Limbic encephalitis associated with anti-Hu antibody induced by atezolizumab treatment for small cell lung cancer: A case report. Oncol Lett. 2026 Mar 17;31(5):177. doi: 10.3892/ol.2026.15532.
28. Nomura Y, Wada Y, Sasaki S, Ikeda T, Ryu T, Takami Y. Autoimmune encephalitis induced by atezolizumab and bevacizumab therapy for advanced hepatocellular carcinoma: a case report. J Med Case Rep. 2026 Jun 18. doi: 10.1186/s13256-026-06037-4.
29. Eun M Y , Hah H , Hwang J .Coexistence of Anti-Hu and Anti-SOX1 Autoantibodies in Atezolizumab-related Encephalitis[J].Journal of the Korean Neurological Association, 2024, 42(1):48-52.DOI:10.17340/jkna.2023.0070.
30. Mandal S , Gosain R .A CASE OF ANTI-HU-ANTIBODIES-MEDICATED ENCEPHALITIS INDUCED BY ATEZOLIZUMAB IN EXTENSIVE-STAGE SMALL CELL LUNG CANCER.Chest, 2021, 160(4):A1524-A1524.DOI:10.1016/j.chest.2021.07.1392.
31. Khachaturyan E A .Atezolizumab-induced encephalitis in a patient with metastatic small cell lung cancer: A clinical case and literature review[J].Journal of Clinical Images and Medical Case Reports, 2024, 5(3).DOI:10.52768/2766-7820/2945.
32. Hirata T , Sugimoto K , Kakegawa T ,et al.A case of suspected autoimmune encephalitis after the introduction of atezolizumab + bevacizumab therapy for hepatocellular carcinoma.Kanzo, 2021, 62(9):590-592.DOI:10.2957/kanzo.62.590.
33. Osuga T , Miyanishi K , Ito R ,et al.Occurrence of Autoimmune Encephalitis in a Patient with Hepatocellular Carcinoma after Atezolizumab and Bevacizumab Combination Therapy: A Case Study.Kanzo, 2023, 64(11):559-566.DOI:10.2957/kanzo.64.559.
34. Li Xiao, Zhang Yanhua. Literature Analysis of Atezolizumab-Induced SOX1 Antibody-Positive Paraneoplastic Encephalitis and Associated Neurological Adverse Events in Patients with Small Cell Lung Cancer. Chin J New Drug. 2023; 32(12): 1269-1274.
35. Chang H, Shin YW, Keam B, Kim M, Im SA, Lee ST. HLA-B27 association of autoimmune encephalitis induced by PD-L1 inhibitor. Ann Clin Transl Neurol. 2020 Nov;7(11):2243-2250. doi: 10.1002/acn3.51213.
